# The Prognostic Value of Neutrophil-to-Lymphocyte Ratio in Patients With Aneurysmal Subarachnoid Hemorrhage: A Systematic Review and Meta-Analysis of Observational Studies

**DOI:** 10.3389/fneur.2021.745560

**Published:** 2021-11-15

**Authors:** Min Shi, Chao Yang, Qing-wen Tang, Ling-fei Xiao, Zu-han Chen, Wen-yuan Zhao

**Affiliations:** ^1^Department of Neurosurgery, Zhongnan Hospital of Wuhan University, Wuhan, China; ^2^Department of Orthopaedics, Zhongnan Hospital of Wuhan University, Wuhan, China; ^3^Institute of Hepatobiliary Diseases of Wuhan University, Zhongnan Hospital of Wuhan University, Wuhan, China

**Keywords:** neutrophil-to-lymphocyte ratio, aneurysmal subarachnoid hemorrhage, prognostic value, poor functional outcome, delayed cerebral ischemia

## Abstract

The neutrophil–to-lymphocyte ratio (NLR), as an essential systemic inflammation factor, has been widely used as a prognostic indicator in various diseases, such as malignant tumors, cardiovascular disease, and intracranial hemorrhage. An increasing number of studies have believed that NLR is a valuable predictor of prognosis for patients with aneurysmal subarachnoid hemorrhage (aSAH). However, these results remain controversial. In the current study, we planned to carry out a systematic review and meta-analysis to investigate the association between NLR and poor outcome, and the occurrence of delayed cerebral ischemia (DCI). We carried out a comprehensive search for published literatures on PubMed, EMBASE, Cochrane Library, and Web of Science databases from inception to April 1, 2021. We conducted an assessment of all included studies based on the principles proposed in the Newcastle-Ottawa Quality Assessment Scale (NOS). Poor outcome and the occurrence of DCI were considered as the main outcome measure. We calculated the pooled odds ratio (OR) and corresponding 95% confidence interval (CI) to examine the strength of the association of NLR with poor outcome or the occurrence of DCI. We strictly selected a total of 10 studies comprising 4,989 patients. Nine studies reported the association between NLR and poor outcome, and five studies reported the association between NLR and the occurrence of DCI. The pooled results indicated higher NLR was significantly associated with both poorer outcomes (OR = 1.32, 95%CI 1.11–1.57; *P* = 0.002, *I*^2^ = 87%), and the occurrence of DCI (OR = 1.72, 95%CI 1.22–2.41; *P* = 0.002, *I*^2^ = 82%) in aSAH patients. The NLR is a valuable indicator of inflammation to independently predict poor outcome and occurrence of DCI after aSAH, where a higher NLR is significantly associated with poor outcomes and occurrence of DCI. These findings suggest that the NLR can help clinicians evaluate the prognosis and identify potentially severe patients early, which may contribute to better management and improve poor prognosis of aSAH patients.

## Introduction

Aneurysmal subarachnoid hemorrhage (aSAH), results from a ruptured intracranial aneurysm, is a serious and life-threatening stroke subtype that can cause damage to the brain and it results in many neurologic disorders, with high mortality and morbidity, as well as severe burdens on the healthcare system (1–3). Despite the mortality of acute subarachnoid hemorrhage (SAH) has gradually dropped from over 50% to ~35% in recent years due to the advances in modern surgical and medical management, SAH remains a significant threat (1, 4).

Early brain injury, cerebral vasospasm or subsequent delayed cerebral ischemia (DCI) are common complications after SAH, which leads to poor neurological outcomes of SAH patients (5, 6). Although the pathogenesis of DCI after aSAH is not completely understood, DCI induced by cerebral vasospasm (CV) after aSAH is considered as a major factor associated with poor functional outcomes and death (7, 8).

There is growing evidence that systemic inflammatory reactions normally occur in the early stage of aSAH, cause damage to the hemorrhagic brain, and influence patients' prognosis (9, 10). Despite the exact mechanism still remains unclear so far, it is well-known that inflammation response plays a critical role in the progression of aSAH by initiating early brain damage, rebleeding, vasospasm, and subsequent DCI (10–12). Simple, quick and sensitive biomarkers are invaluable to aSAH therapy than most biomarkers that require complicated cerebrospinal fluid sample collection and laboratory tests (13).

The neutrophil-to-lymphocyte ratio (NLR), as a ratio of the absolute neutrophil count and absolute lymphocyte count in the peripheral blood, is a simple, readily accessible, inexpensive marker of systemic inflammation (14). Over the past decade, NLR has been demonstrated to be a prognostic biomarker for cardiovascular diseases, cancers, ischemic stroke, infectious and inflammatory diseases (9, 15–17). There has been a recent surge in research investigating the prognostic value of high NLR in prognosis of patients with aSAH. However, the association between NLR and clinical prognosis remains controversial. In the current study, we therefore systematically reviewed and performed a meta-analysis to quantitatively analyze the existing evidence and evaluate the prognostic value of NLR in aSAH patients.

## Materials and Methods

We finished the meta-analysis following the Preferred Reporting Items for Systematic Reviews and Meta-Analyses (PRISMA statement) guidelines (18). The result of PRISMA checklist was showed in Supplementary Data Sheet 1.

### Search Strategy

We carried out a comprehensive search for published literatures on PubMed, EMBASE, Cochrane Library, and Web of Science databases from inception to April 1, 2021. The search keywords used for each database were as follows: (“subarachnoid hemorrhage” OR “SAH” OR “ruptured Brain aneurysm” OR “ruptured cerebral aneurysm” OR “ruptured intracranial aneurysm”) AND (“neutrophil-to-lymphocyte ratio” OR “neutrophil lymphocyte ratio” OR “neutrophil to lymphocyte ratio” OR “neutrophil/Lymphocyte ratio” OR “NLR”). The free terms are presented in Supplementary Data Sheet 2.

### Inclusion and Exclusion Criteria

The articles were required to satisfy the following inclusion criteria: (1) included adult patients (≥18 years of age) diagnosed with aSAH; (2) studies showing an association between NLR and aSAH; and (3) the odds ratio (OR) and 95% confidence interval (CI) for efficacy outcome were provided from the multivariate analysis.

Articles were excluded if they met the following criteria: (1) not written in English; (2) non-human subjects; (3) patients not diagnosed as with aSAH; (4) subjects not excluded for infection, cancer, severe kidney or liver disease, history of auto-immune or hematologic disorder; (5) studies only providing data from unadjusted univariate analysis; and (6) incomplete or deficient data.

### Outcome Measure

For the analyses, the two main outcome measures were poor function outcomes and the occurrence of DCI.

Poor function outcome was defined as GOS score 1 to 3 or mRS score 3 to 6 at the end of follow-up.

DCI was defined as the development of focal clinical deterioration (such as hemiparesis, aphasia, apraxia, or neglect), or a drop of at least 2 points on Glasgow Coma Scale, which was not obvious immediately after aneurysm surgery and cannot be attributed to other causes by means of clinical assessment, imaging, and appropriate laboratory studies.

### Study Selection, Data Extraction, and Quality Assessment

The two authors (MS and CY) independently searched and identified the relevant literary works according to eligibility criteria. The following data were extracted by using pre-defined checklist: first author, publication year, country, study design, number of patients, sex ratio and median age of patients, research method, sample time, optimal cut-off value of NLR, and follow-up period. Any disagreements were resolved through a discussion among the researchers or obtaining an opinion from a third reviewer. When we encountered important data loss, we attempted to contact the original author to retrieve the data.

We used the Newcastle-Ottawa scale (NOS) to evaluate the quality of the included studies including the following domains: selection, comparability, and outcome of interest (19). Any disagreement encountered in the process was resolved by discussion or with the third reviewer. The total score ranged from 0 to 9 with higher scores indicating higher quality; studies with scores ≥ 6 were regarded as high quality (Supplementary Table 1).

### Statistical Analysis

We finished the data analysis using the Review Manager (RevMan) software (version 5.4.1, Copenhagen: The Nordic Cochrane Center, Cochrane Collaboration). Extracted odds ratio (OR) and corresponding 95% confidence interval (CI) were pooled and weighted using a generic inverse-variance method to evaluate the strength of the association of NLR with poor outcome or the occurrence of DCI. Heterogeneity among the studies was evaluated by using the *I*^2^ statistic and Cochrane *Q*-statistic test. When *I*^2^ > 50% or *P* < 0.10 indicated the presence of heterogeneity, we chose the random-effect model, and the fixed-effect model was chosen if *I*^2^ <50% or *P* > 0.10. We also carried out subgroup and sensitivity analyses to identify and minimize heterogeneity. Publication bias was evaluated by visually inspecting funnel plots.

## Results

### Study Selection

**Figure 1:**
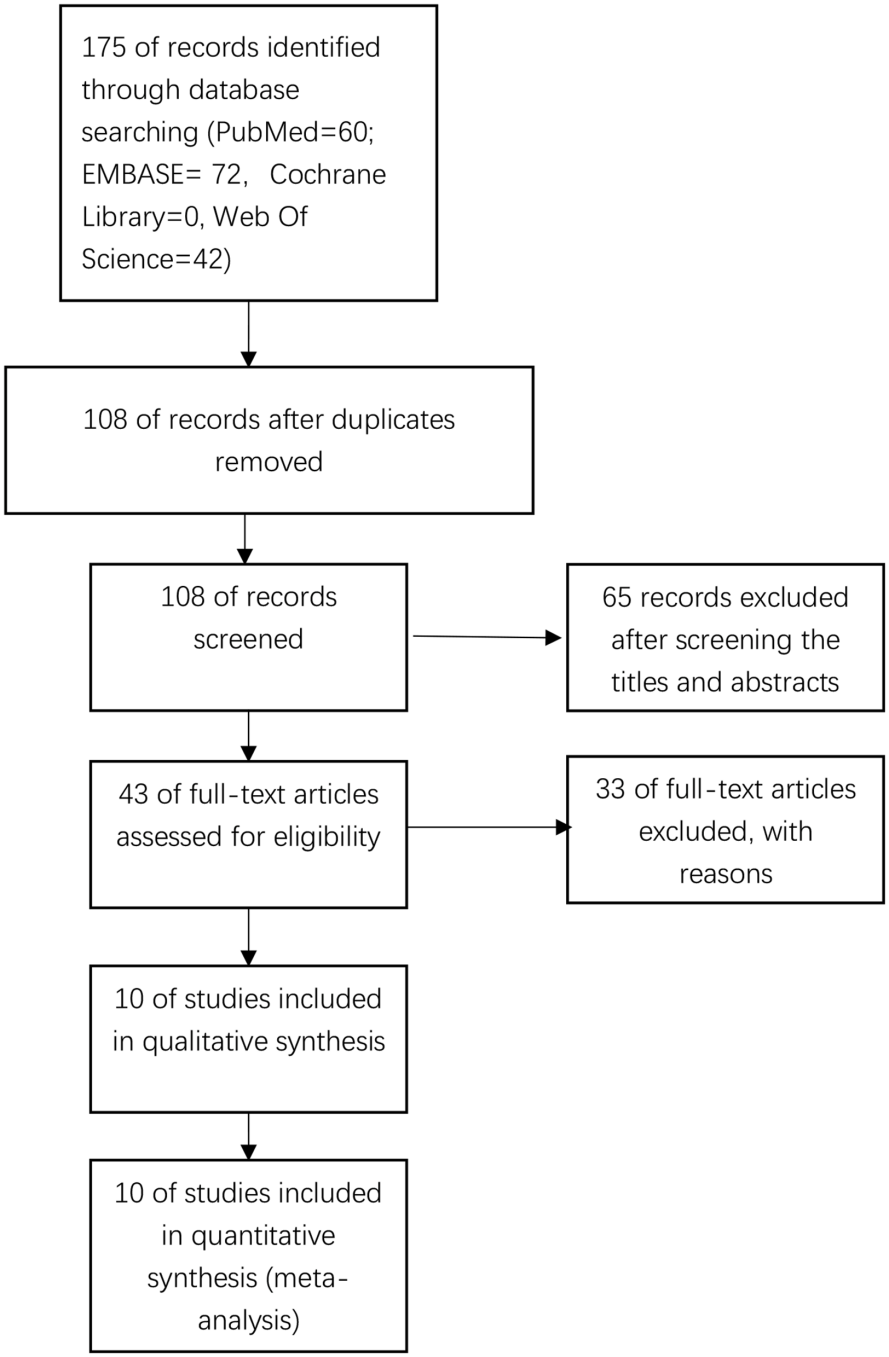
Flow diagram of the study selection process.

We systematically searched total 175 studies in the initial literature search. After the exclusion of duplicates, 108 studies remained. By screening the titles and abstracts, 65 studies were removed and only 43 studies were left. Thirty-two studies were excluded according to full-text analysis. Finally, we included a total of 10 studies in the current study. Figure 1 shows the flow diagram of the study screening process.

### Study Characteristics

The characteristics of the included studies are summarized in Table 1. We included 10 studies consisting of 4,140 patients in this meta-analysis (10, 20–28). Nine studies investigated the association between NLR and poor functional outcome (10, 20–24, 26–28), and six studies investigated the association between NLR and DCI (20, 22, 25–27). Most of the studies were retrospective observational studies, and only three studies were prospective studies. Eight studies were conducted in Asia (six of them were China, while two of them were Korea), two in USA, and one in Germany. The number of patients varied from 178 to 1,067. Among the studies included, most studies collected blood samples on admission. The follow-up period was mostly 3 months. The cut-off values of NLR varied from 4.0 to 14.0, except for three studies that did not report the cut-off value. All of the included studies received a NOS score ≥ 6, which represented high quality.

**Table 1:** Baseline characteristics of the included studies.

**References**	**Design**	**Country**	**Duration**	**Number**	**Mean age**	**Sex (M/F)**	**Sample time**	**Outcome measure**	**Follow up**	**Cut-off value**
Yun et al. (10)	R	Korea	2012–2021	680	56.9 ± 13.4^a^ 55.2 ± 12.4^b^	216/464	Admission	Poor outcome^*^	3 months	4.0
Zhang et al. (20)	R	China	2013–2016	532	54.0 ± 10.5	98/221	Admission	Poor outcome^*^/DCI^*^	3–6 months	4.0
Chen and Zhang (21)	R	China	2015–2019	262	58 (49–65)^a^ 63 (54–69)^b^	100/162	Admission	Poor outcome^*^	3 months	NR
Xiang (28)	R	China	2016–2018	235	58.10 ± 9.97	93/142	NR	Poor outcome^*^	3 months	NR
Yi et al. (22)	R	Korea	2012–2020	498	56.9 ± 13.4^a^ 65.2 ± 15.7^b^	162/336	Admission	Poor outcome^*^/DCI^*^	3 months	5.7
Zhang et al. (23)	R	China	2015–2017	178	57.64 ± 10.23	62/116	Admission	Poor outcome^*^	3 months	NR
Giede-Jeppe et al. (24)	R	German	2008–2012	319	51 (43–59)^a^ 57 (49–70)^b^	98/221	Admission	Poor outcome^*^	12 months	7.05
Wu et al. (25)	R	China	2015, 1–12	122	55.3 ± 10.6	48/74	Admission	DCI^*^	Discharge	11.47
Al-Mufti et al. (26)	P	USA	2006–2015	1,067	>53 y (55%)	336/731	Within 24 h	Poor outcome^*^/DCI^*^	3 months	5.9
Tao et al. (27)	P	China	2014–2015	247	55.9 ± 11.9	88/159	Admission	Poor outcome^*^/DCI^*^	3 months	14.0

a *Age for good outcome patients*.

b *Age for poor outcome patients*.

* *Variables are calculated by multivariable analysis*.

*M, male; F, female; R, retrospective; P, prospective; NR, not reported; DCI, delayed cerebral ischemia*.

### Association Between NLR and Poor Functional Outcome

**Figure 2:**
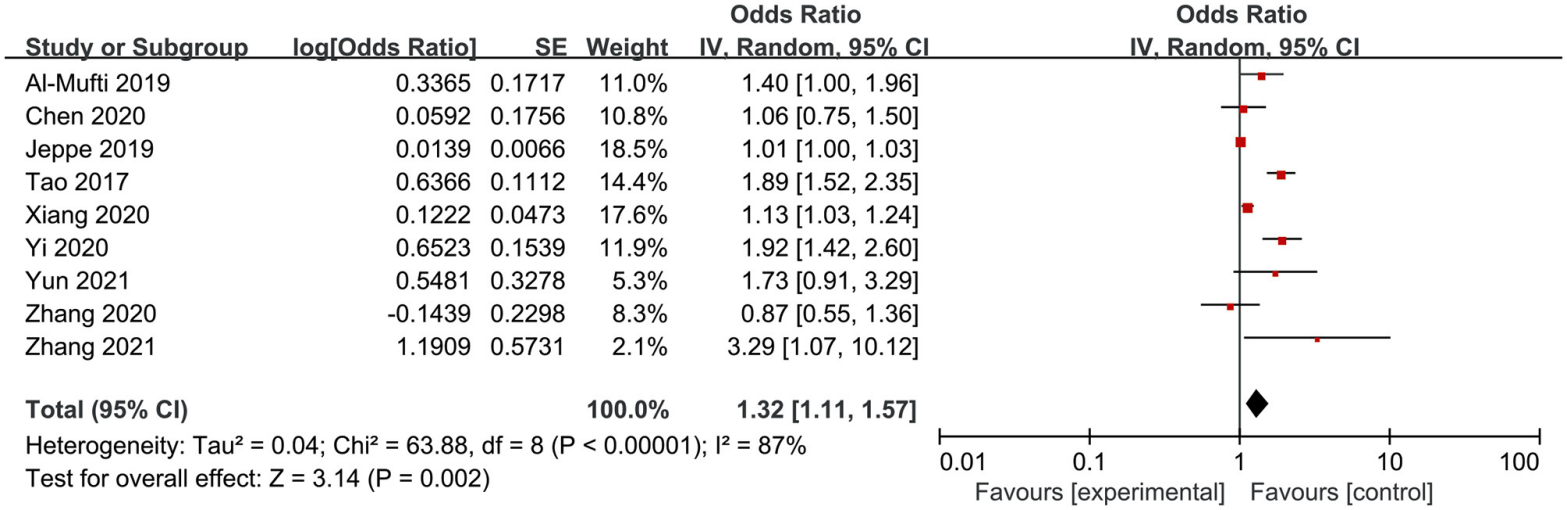
Meta-analysis of the association between NLR and poor outcome.

Nine studies involving 4,018 participants investigated the association between NLR and poor functional outcome in patients of aSAH. The pooled OR indicated that a higher NLR was significantly associated with poor functional outcome at the end up of follow up (OR = 1.32, 95%CI 1.11–1.57; *P* = 0.002), with significant heterogeneity (*I*^2^ = 87%, *P* < 0.00001) (Figure 2).

### Association Between NLR and the Occurrence of DCI

**Figure 3:**
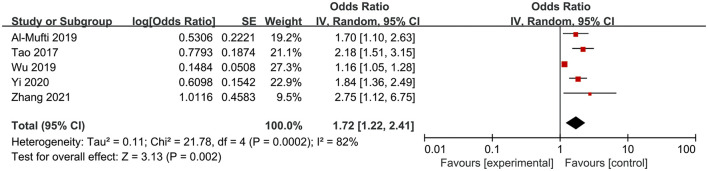
Meta-analysis of the association between NLR and the occurrence of DCI.

Six studies involving 2,466 participants investigated the association between NLR and the occurrence of DCI in patients of aSAH. The pooled OR indicated that a high NLR was significantly associated with the occurrence of DCI (OR = 1.72, 95%CI 1.22–2.41; *P* = 0.002), with significant heterogeneity (*I*^2^ = 82%, *P* = 0.0002) (Figure 3).

### Subgroup Analysis of Poor Outcome

We performed a series of subgroup analyses based on study design, ethnicity, pre-defined sample size (size ≥ 400 and size <400) and NLR cutoff value to investigate other factors that could affect poor functional outcomes of NLR in aSAH patients (Table 2). In the subgroup analysis according to study design, higher NLR was significantly associated with poor outcome in the retrospective study (OR = 1.19, 95%CI 1.02–1.40; *P* = 0.03) and prospective group (OR = 1.67, 95%CI 1.25–2.23; *P* = 0.0005). The same analysis conducted on the basis of ethnicity showed that high NLR was significantly associated with poor outcome in Asian (OR = 1.42, 95%CI 1.08–1.87; *P* = 0.01), but not non-Asian samples (OR = 1.14, 95%CI 0.84–1.54; *P* = 0.40). According to sample size, high NLR was significantly associated with poor outcomes in large sample sizes (OR = 1.72, 95%CI 1.37–2.14; *P* < 0.00001); however, high NLR was not associated with poor outcomes in small sample sizes (OR = 1.17, 95%CI 0.84–1.54; *P* = 0.40). According to different NLR cutoff value, high NLR was significantly associated with poor outcomes in lower NLR cutoff value subgroup (OR = 1.72, 95%CI 1.37–2.14; *P* < 0.00001), with no significant heterogeneity (*I*^2^ = 7%, *P* = 0.36); however, high NLR was not associated with poor outcomes in higher NLR cutoff value subgroup (OR = 1.37, 95%CI 0.74–2.52; *P* = 0.31), with significant heterogeneity (*I*^2^ = 97%, *P* < 0.00001). In our subgroup analysis, no significant heterogeneity was observed in the lower NLR cutoff value subgroup (*I*^2^ = 7%, *P* = 0.36). It suggests that NLR cutoff value may be the potential sources of heterogeneity.

**Table 2:** Subgroup analysis of the association of NLR with poor outcome and DCI.

**Subgroup**	**No. of studies**	***I* ^**2**^**	***P-*value**	**Model**	**OR (95%CI)**	***P-*value**
**NLR and poor outcome**						
Total	9	87%	<0.00001	Random	1.32 (1.11–1.57)	0.002
**Ethnicity**						
Asian	7	82%	<0.00001	Random	1.42 (1.08–1.87)	0.01
Non-Asian	2	72%	0.06	Random	1.14 (0.84–1.54)	0.40
**Sample size**						
>400	4	7%	0.36	Random	1.72 (1.37–2.14)	<0.00001
<400	5	89%	<0.00001	Random	1.17 (0.98–1.40)	0.08
**Study design**						
Retrospective	7	80%	<0.0001	Random	1.19 (1.02–1.40)	0.03
Prospective	2	54%	0.14	Random	1.67 (1.25–2.23)	0.0005
**Cut-off value**						
<6.8	4	7%	0.36	Random	1.72 (1.37–2.14)	<0.00001
≥6.8	2	97%	<0.00001	Random	1.37 (0.74–2.52)	0.31
**NLR and DCI**						
Total	5	82%	0.0002	Random	1.72 (1.22–2.41)	0.002
**Ethnicity**						
Asian	4	85%	0.0002	Random	1.74 (1.16–2.61)	0.008
Non-Asian	1	-	-	Random	1.70 (1.10–2.63)	0.02
**Sample size**						
>400	3	0%	0.64	Random	1.85 (1.46–2.35)	<0.00001
<400	2	91%	0.001	Random	1.55 (0.84–2.87)	0.16
**Study design**						
Retrospective	3	82%	0.004	Random	1.59 (1.02–2.48)	0.04
Prospective	2	0%	<0.00001	Random	1.97 (1.48–2.60)	<0.00001

*OR, odds ratio; CI, confidence interval; NLR, neutrophil-to-lymphocyte ratio; DCI, delayed cerebral ischemia*.

### Sensitivity Analysis and Publication Bias

We conducted a sensitivity analysis to test for heterogeneity using the leave-one-out approach. The results of these analyses were showed in Supplementary Table 2. For the association between NLR and poor outcome, the results of the sensitivity analysis were consistent with the overall results, and heterogeneity was not obviously altered by removing any studies. For the association between NLR and DCI, one study (Wu et al.) is the major source of the heterogeneity. Heterogeneity was substantially decreased after removing this study (*I*^2^ = 0, *P* = 0.70), but removing this study did not change the overall pattern of results (OR = 1.94, 95%CI 1.59–2.37; *P* < 0.00001).

**Figure 4:**
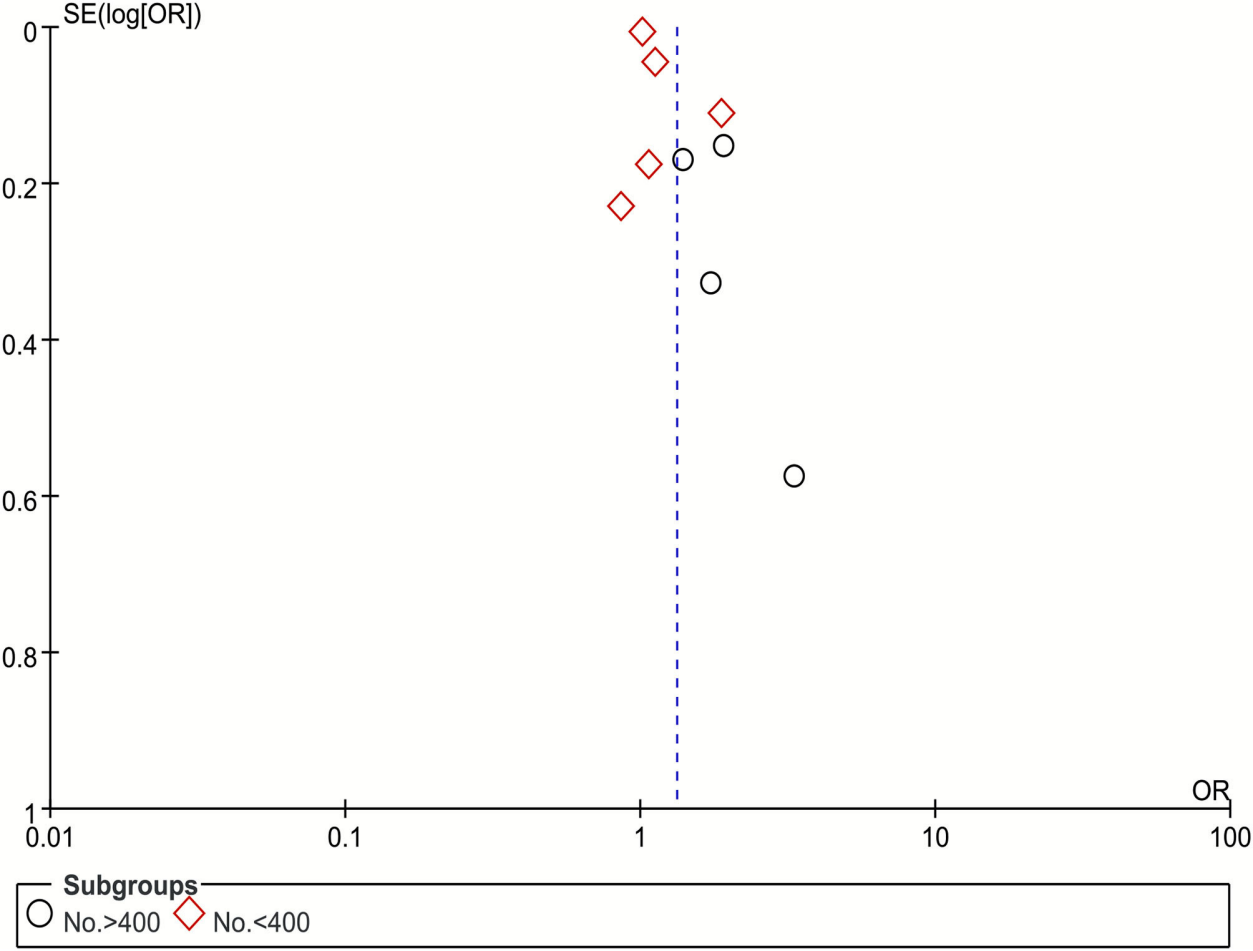
Funnel plot of publication bias of NLR with poor outcome.

We produced a funnel plot for the poor outcome to assess the publication bias, and observed no obvious publication bias (Figure 4).

## Discussion

In this systematic review and meta-analysis, we primarily evaluated the prognostic value of NLR in the poor functional outcome and the occurrence of the DCI in aSAH patients. We included 10 studies consisting of 4,989 patients and found that nine studies assessed the association of NLR and poor functional outcome, while five assessed the association of NLR and the occurrence of DCI. The pooled results suggested that higher NLR is significantly associated with poor functional outcomes and the occurrence of DCI.

SAH has been identified as a devastating subtype of hemorrhagic stroke with high mortality and disability (29). Despite advancements in the treatment and management of SAH patients, the prognosis is still extremely poor (30). It is therefore urgent to develop a valuable prognostic indicator for evaluation of the prognosis in patients with aSAH. Although many biomarkers have been reported as predictors to predict prognosis, most of them are not applicable for clinical use. Laboratory biomarkers have attracted considerable attention owing to their practicality and sensitivity; however, most biomarkers require special laboratory equipment or complex sample collection of which may increase additional risk of complications and limit the application (31).

In recent years, the relationship between inflammatory response and subarachnoid hemorrhage has been widely studied. Systemic inflammation is considered an important pathophysiological mechanism in aSAH. A substantial number of inflammatory pathways have been shown to be activated in the disease, although the specific mechanisms are not fully understood (32). The introduction of blood into the subarachnoid space leads to a systemic inflammatory response syndrome that results in increased neutrophils and decreased lymphocytes, and both correlate with the severity of the tissue inflammation and hemorrhagic irritation since they are related to the abrupt release of large amount of endogenous catecholamines, corticosteroids, or other cytokines (33).

Studies have suggested that neutrophils can stimulate and inflammatory responses, while lymphocytes play an important role in anti-inflammatory and endothelial protective functions (34). NLR represents the balance between innate immunity (neutrophil) and adaptive immunity (lymphocyte) (35). Therefore, elevated NLR indicates worse prognosis in aSAH patients. NLR is an emerging field of study especially in aSAH research; however, its utility as a predictor for functional outcome and DCI remains controversial. Many studies support the idea that NLR can predict poor outcomes and the occurrence of DCI; however, some have failed to show such an association (10, 21, 23), necessitating an objective meta-analytic approach.

This is the first meta-analysis that focuses on the association between NLR and poor outcomes and DCI. All of the included studies were considered high quality and were adjusted with potential confounding factors. The results of sensitivity analysis and subgroup analysis by study design, ethnicity, pre-defined sample size and NLR cutoff value were generally consistent with the overall analysis. However, we observed substantial heterogeneity in the current study. For the poor outcome, subgroup analysis based on cut-off value indicated that NLR cutoff value might be the source of heterogeneity. For DCI, we determined that Wu's study was the source of this heterogeneity. After excluding the study, the overall result did not significantly change. Therefore, we have reason to believe that our data are reliable and that the findings can be valuable for clinical application.

Although NLR was significantly associated with poor function outcome in our study, we found no significant association between NLR and functional outcome in non-Asian groups, small sample sizes and high NLR cutoff value group. Several reasons may exist for these findings. First, only two studies were included in the non-Asian group, which can reduce the statistical power and lead to unreliable results. Second, some studies observed positive correlations between NLR levels and ethnicity in healthy populations; it may therefore be the case that NLR has various predictive abilities for poor functional outcome across ethnicity (36). Moreover, it is likely that small sample sizes limit the detection power of significant effects, and it is necessary for future studies to consider larger sample sizes. Interestingly, we found a greater magnitude of effect on poor outcome in lower NLR cutoff value group. It suggested that the prognostic predictive ability of the NLR might be affected by cutoff values.

For the patients with aSAH, it is necessary to identify potentially severe patients early, in order to facilitate transfer to intensive care and effective time management (37). Currently, the most commonly used clinical predictive models for identifying aSAH patients with unfavorable prognoses are mainly the Hunt-Hess scale and WFNS (38). However, these prognostic models are solely based on clinical information and ignore the predictive value of biological elements. Our findings address this gap in the field and guide future research regarding the prognostic value of NLR. DCI is a major cause of poor outcome and even death, but the strongest predictors are severity of SAH as detected by CT scans and poor neurological grades (1, 6). Our results indicated that patients with high admission NLR value may have a higher risk of DCI and poor prognosis. Clinicians should therefore be vigilant about these patients and treat them the earliest opportunity. While our study assessed NLR on admission, this measure only represents admission status, and more studies are needed which combine established risk factors and prognostic markers to establish a schema for clinical guidance.

There were also some limitations to this study. First, only 10 studies were eligible for inclusion, and the number of studies included for individual outcome was smaller. Second, the majority of included studies were small samples, single center, and retrospective, which inevitably affected the reliability of the results. Third, there was significant heterogeneity in each outcome measure. Fourth, despite adjusting for potential confounding factors, there are still some potential factors such as the initial severity of ictus, that we did not assess but may nevertheless influence the results. In the future, we need to design more prospective, multicenter, large-sample clinical trials to investigate the prognostic value of NLR in aSAH.

## Conclusion

The NLR is a valuable indicator of inflammation to independently predict poor outcome and occurrence of DCI after aSAH, where a higher NLR is significantly associated with poor outcomes and occurrence of DCI. These findings suggest that the NLR may help clinicians evaluate the prognosis and identify potentially severe patients early, which may contribute to better management and improve poor prognosis of aSAH patients.

## Data Availability Statement

The original contributions presented in the study are included in the article/Supplementary Material, further inquiries can be directed to the corresponding author/s.

## Author Contributions

MS: took responsibility for the integrity of the data and the accuracy of the data analysis. MS and CY: contribute significantly to data analyses and manuscript preparation. MS, QT, ZC, and LX: made critical revision of the manuscript for important intellectual content. WZ: supervision. All authors contribute to the conception, design, analysis and interpretation of data of the study.

## Funding

This study was funded by the Key Research and Development Plan of Hubei Science and Technology Department. No. 2020BCB033.

## Conflict of Interest

The authors declare that the research was conducted in the absence of any commercial or financial relationships that could be construed as a potential conflict of interest.

## Publisher's Note

All claims expressed in this article are solely those of the authors and do not necessarily represent those of their affiliated organizations, or those of the publisher, the editors and the reviewers. Any product that may be evaluated in this article, or claim that may be made by its manufacturer, is not guaranteed or endorsed by the publisher.

## References

[1] Macdonald RL Schweizer TA. Spontaneous subarachnoid haemorrhage. Lancet. (2017) 389:655–66. 10.1016/S0140-6736(16)30668-7 27637674

[2] de Rooij NK Linn FHH van der Plas JA Algra A Rinkel GJE. Incidence of subarachnoid haemorrhage: a systematic review with emphasis on region, age, gender and time trends. J Neurol Neurosurg Psychiatry. (2007) 78:1365–72. 10.1136/jnnp.2007.117655 17470467 PMC2095631

[3] Kundra S Mahendru V Gupta V Choudhary AK. Principles of neuroanesthesia in aneurysmal subarachnoid hemorrhage. J Anaesthesiol Clin Pharmacol. (2014) 30:328–37. 10.4103/0970-9185.137261 25190938 PMC4152670

[4] Petridis AK Kamp MA Cornelius JF Beez T Beseoglu K Turowski B. Aneurysmal Subarachnoid Hemorrhage. Dtsch Arztebl Int. (2017) 114:226–36. 10.3238/arztebl.2017.0226 28434443 PMC5624452

[5] de Oliveira Manoel AL Goffi A Marotta TR Schweizer TA Abrahamson S Macdonald RL. The critical care management of poor-grade subarachnoid haemorrhage. Crit Care. (2016) 20:21. 10.1186/s13054-016-1193-9 26801901 PMC4724088

[6] Rowland MJ Hadjipavlou G Kelly M Westbrook J Pattinson KTS. Delayed cerebral ischaemia after subarachnoid haemorrhage: looking beyond vasospasm. Br J Anaesth. (2012) 109:315–29. 10.1093/bja/aes264 22879655

[7] Keyrouz SG Diringer MN. Clinical review: prevention and therapy of vasospasm in subarachnoid hemorrhage. Crit Care. (2007) 11:220. 10.1186/cc5958 17705883 PMC2206512

[8] Dankbaar JW Rijsdijk M van der Schaaf IC Velthuis BK Wermer MJH Rinkel GJE. Relationship between vasospasm, cerebral perfusion, and delayed cerebral ischemia after aneurysmal subarachnoid hemorrhage. Neuroradiology. (2009) 51:813–9. 10.1007/s00234-009-0575-y 19623472 PMC2773037

[9] Lattanzi S Brigo F Trinka E Cagnetti C Di Napoli M Silvestrini M. Neutrophil-to-lymphocyte ratio in acute cerebral hemorrhage: a system review. Transl Stroke Res. (2019) 10:137–45. 10.1007/s12975-018-0649-4 30090954

[10] Yun S Yi HJ Lee DH Sung JH. Systemic inflammation response index and systemic immune-inflammation index for predicting the prognosis of patients with aneurysmal subarachnoid hemorrhage. J Stroke Cerebrovasc Dis. (2021) 30:105861. 10.1016/j.jstrokecerebrovasdis.2021.105861 34034125

[11] Dumont AS Dumont RJ Chow MM Lin C-L Calisaneller T Ley KF. Cerebral vasospasm after subarachnoid hemorrhage: putative role of inflammation. Neurosurgery. (2003) 53:123–33. 10.1227/01.NEU.0000068863.37133.9E 12823881

[12] Fu C Yu W Sun L Li D Zhao C. Early cerebral infarction following aneurysmal subarachnoid hemorrhage: frequency, risk factors, patterns, and prognosis. Curr Neurovasc Res. (2013) 10:316–24. 10.2174/15672026113109990027 24016219

[13] Wang J-Y Zhang X-T Wang J-Q Wang C-Y Zheng W-L Pan Z-M. Admission neutrophil-lymphocyte ratio predicts rebleeding following aneurismal subarachnoid hemorrhage. World Neurosurg. (2020) 138:e317–e22. 10.1016/j.wneu.2020.02.112 32112936

[14] Akalin Ç Altaş H Akçay Çelik M. White blood count can be a practical guide for the differential diagnosis of breast abscess and idiopathic granulomatous mastitis. Cureus. (2020) 12:e10468. 10.7759/cureus.10468 33083171 PMC7566977

[15] Afari ME Bhat T. Neutrophil to lymphocyte ratio (NLR) and cardiovascular diseases: an update. Expert Rev Cardiovasc Ther. (2016) 14:573–7. 10.1586/14779072.2016.1154788 26878164

[16] Mitsuya K Nakasu Y Kurakane T Hayashi N Harada H Nozaki K. Elevated preoperative neutrophil-to-lymphocyte ratio as a predictor of worse survival after resection in patients with brain metastasis. J Neurosurg. (2017) 127:433–7. 10.3171/2016.8.JNS16899 27911233

[17] de Jager CPC van Wijk PTL Mathoera RB de Jongh-Leuvenink J van der Poll T Wever PC. Lymphocytopenia and neutrophil-lymphocyte count ratio predict bacteremia better than conventional infection markers in an emergency care unit. Crit Care. (2010) 14:R192. 10.1186/cc9309 21034463 PMC3219299

[18] Moher D Liberati A Tetzlaff J Altman DG. Preferred reporting items for systematic reviews and meta-analyses: the PRISMA statement. Ann Intern Med. (2009) 151:264–9. 10.7326/0003-4819-151-4-200908180-00136 19622511

[19] Stang A. Critical evaluation of the Newcastle-Ottawa scale for the assessment of the quality of nonrandomized studies in meta-analyses. Eur J Epidemiol. (2010) 25:603–5. 10.1007/s10654-010-9491-z 20652370

[20] Zhang B Lin L Yuan F Song G Chang Q Wu Z. Clinical application values of neutrophil-to-lymphocyte ratio in intracranial aneurysms. Aging (Albany NY). (2021) 13:5250–62. 10.18632/aging.202445 33526720 PMC7950281

[21] Chen L Zhang Q. Increased mean platelet volume is associated with poor outcome in patients with aneurysmal subarachnoid hemorrhage. World Neurosurg. (2020) 137:e118–25. 10.1016/j.wneu.2020.01.068 31954895

[22] Yi HJ Lee DH Sung JH. Inflammation-based Scores are associated with the prognosis of patients with aneurysmal subarachnoid hemorrhage after neuro-intervention. Curr Neurovasc Res. (2020) 17:676–85. 10.2174/1567202617999201117120905 33208077

[23] Zhang P Li Y Zhang H Wang X Dong L Yan Z. Prognostic value of the systemic inflammation response index in patients with aneurismal subarachnoid hemorrhage and a Nomogram model construction. Br J Neurosurg. (2020) 1–7. 10.1080/02688697.2020.1831438 33044089

[24] Giede-Jeppe A Reichl J Sprügel MI Lücking H Hoelter P Eyüpoglu IY. Neutrophil-to-lymphocyte ratio as an independent predictor for unfavorable functional outcome in aneurysmal subarachnoid hemorrhage. J Neurosurg. (2019) 132:400–7. 10.3171/2018.9.JNS181975 30717052

[25] Wu Y He Q Wei Y Zhu J He Z Zhang X. The association of neutrophil-to-lymphocyte ratio and delayed cerebral ischemia in patients with aneurysmal subarachnoid hemorrhage: possible involvement of cerebral blood perfusion. Neuropsychiatr Dis Treat. (2019) 15:1001–7. 10.2147/NDT.S190477 31118639 PMC6499147

[26] Al-Mufti F Amuluru K Damodara N Dodson V Roh D Agarwal S. Admission neutrophil-lymphocyte ratio predicts delayed cerebral ischemia following aneurysmal subarachnoid hemorrhage. J Neurointerv Surg. (2019) 11:1135–40. 10.1136/neurintsurg-2019-014759 30979846

[27] Tao C Wang J Hu X Ma J Li H You C. Clinical value of neutrophil to lymphocyte and platelet to lymphocyte ratio after aneurysmal subarachnoid hemorrhage. Neurocrit Care. (2017) 26:393–401. 10.1007/s12028-016-0332-0 28028791

[28] Lai X Zhang W Ye M Liu X Luo X. Development and validation of a predictive model for the prognosis in aneurysmal subarachnoid hemorrhage. J Clin Lab Anal. (2020) 34:e23542. 10.1002/jcla.23542 32860455 PMC7755773

[29] Adil SM Liu B Charalambous LT Kiyani M Gramer R Swisher CB. Healthcare economics of hydrocephalus after aneurysmal subarachnoid hemorrhage in the United States. Transl Stroke Res. (2019) 10:650–63. 10.1007/s12975-019-00697-9 30864050

[30] Venti M. Subarachnoid and intraventricular hemorrhage. Front Neurol Neurosci. (2012) 30:149–53. 10.1159/000333625 22377884

[31] Ma X Lan F Zhang Y. Associations between C-reactive protein and white blood cell count, occurrence of delayed cerebral ischemia and poor outcome following aneurysmal subarachnoid hemorrhage: a systematic review and meta-analysis. Acta Neurol Belg. (2021) 121:1311–24. 10.1007/s13760-020-01496-y 33423218 PMC7796813

[32] Khey KMW Huard A Mahmoud SH. Inflammatory pathways following subarachnoid hemorrhage. Cell Mol Neurobiol. (2020) 40:675–93. 10.1007/s10571-019-00767-4 31808009 PMC11448815

[33] Nóbrega Lima Rodrigues de Morais A Ribeiro Baylão VM Martins Silva T Gomes Dos Santos A Azevedo M de Oliveira AJM. Is neutrophil-lymphocyte ratio a useful tool for predicting outcome in subarachnoid hemorrhage? A systematic review. Neurosurg Rev. (2021). 10.1007/s10143-021-01484-7 33587200

[34] Imtiaz F Shafique K Mirza SS Ayoob Z Vart P Rao S. Neutrophil lymphocyte ratio as a measure of systemic inflammation in prevalent chronic diseases in Asian population. Int Arch Med. (2012) 5:2. 10.1186/1755-7682-5-2 22281066 PMC3277482

[35] Guo Z Yu S Xiao L Chen X Ye R Zheng P. Dynamic change of neutrophil to lymphocyte ratio and hemorrhagic transformation after thrombolysis in stroke. J Neuroinflammation. (2016) 13:199. 10.1186/s12974-016-0680-x 27561990 PMC5000487

[36] Azab B Camacho-Rivera M Taioli E. Average values and racial differences of neutrophil lymphocyte ratio among a nationally representative sample of United States subjects. PLoS ONE. (2014) 9:e112361. 10.1371/journal.pone.0112361 25375150 PMC4223021

[37] Udy AA Vladic C Saxby ER Cohen J Delaney A Flower O. Subarachnoid hemorrhage patients admitted to intensive care in Australia and New Zealand: a multicenter cohort analysis of in-hospital mortality over 15 years. Crit Care Med. (2017) 45:e138–e45. 10.1097/CCM.0000000000002059 27749342

[38] Aggarwal A Dhandapani S Praneeth K Sodhi HBS Pal SS Gaudihalli S. Comparative evaluation of H&H and WFNS grading scales with modified H&H (sans systemic disease): a study on 1000 patients with subarachnoid hemorrhage. Neurosurg Rev. (2018) 41:241–7. 10.1007/s10143-017-0843-y 28299469

