# Supplementary material for: The Prognostic Value of Neutrophil-to-Lymphocyte Ratio in Patients With Aneurysmal Subarachnoid Hemorrhage: A Systematic Review and Meta-Analysis of Observational Studies
Source: Front Neurol. 2021 Nov 15;12:745560. doi: 10.3389/fneur.2021.745560 (PMC8636120; doi:10.3389/fneur.2021.745560)
Supplement: Supplementary file 1 [file Table_1.DOCX]

Supplementary table 1: Quality assessment of included studies using the Newcastle Ottawa Scale for cohort studies.

| Study | Selection | | | | Comparability | Outcome | | | Totals |
| --- | --- | --- | --- | --- | --- | --- | --- | --- | --- |
|  | a | b | c | d | e | f | g | h |  |
| Yun  2021 | 1 | 1 | 1 | 0 | 2 | 1 | 1 | 1 | 8 |
| Zhang 2021 | 1 | 1 | 1 | 0 | 1 | 1 | 1 | 1 | 7 |
| Chen 2020 | 1 | 1 | 1 | 0 | 2 | 1 | 1 | 1 | 8 |
| Xiang 2020 | 1 | 1 | 1 | 0 | 2 | 1 | 1 | 1 | 8 |
| Yi  2020 | 1 | 1 | 1 | 0 | 2 | 1 | 1 | 1 | 8 |
| Zhang  2020 | 1 | 1 | 1 | 0 | 1 | 1 | 1 | 1 | 7 |
| Jeppe 2019 | 1 | 1 | 1 | 0 | 2 | 1 | 1 | 1 | 8 |
| Wu  2019 | 1 | 1 | 1 | 0 | 2 | 1 | 0 | 1 | 7 |
| Al-Mufti 2019（2） | 1 | 1 | 1 | 0 | 2 | 1 | 1 | 1 | 8 |
| Al-Mufti 2019（1） | 1 | 1 | 1 | 0 | 1 | 1 | 1 | 1 | 7 |
| Tao 2017 | 1 | 1 | 1 | 0 | 2 | 1 | 1 | 1 | 8 |

**Selection**: a: Representativeness of the exposed cohort; b: Selection of the non-exposed cohort; c: Ascertainment of exposure; D: Demonstration that outcome of interest was not present at start of study

**Comparability**: e: Comparability of Cohorts on Basis of Design or Analysis Time to Follow-Up

**Outcome**: f: Assessment of outcome; g: Follow-up long enough for outcomes to occur; h: Adequacy of follow-up of cohorts
