# Supplementary material for: The Prognostic Value of Neutrophil-to-Lymphocyte Ratio in Patients With Aneurysmal Subarachnoid Hemorrhage: A Systematic Review and Meta-Analysis of Observational Studies
Source: Front Neurol. 2021 Nov 15;12:745560. doi: 10.3389/fneur.2021.745560 (PMC8636120; doi:10.3389/fneur.2021.745560)
Supplement: Supplementary file 2 [file Table_2.DOCX]

Supplementary table 2: Sensitivity analysis of NLR with poor functional outcome and DCI

| Removed study | OR | 95％CI | Z | P | I^2^ |
| --- | --- | --- | --- | --- | --- |
| NLR for poor functional outcome | | | | | |
| Al-Mufti 2019（2） | 1.31 | 1.09-1.58 | 2.88 | 0.004 | 88 |
| Chen 2020 | 1.36 | 1.13-1.64 | 3.19 | 0.001 | 89 |
| Jeppe 2019 | 1.42 | 1.12-1.79 | 2.88 | 0.004 | 79 |
| Tao 2017 | 1.22 | 1.05-1.42 | 2.54 | 0.01 | 79 |
| Xiang 2020 | 1.40 | 1.06-1.85 | 2.35 | 0.02 | 88 |
| Yi 2020 | 1.25 | 1.05-1.48 | 2.58 | 0.01 | 85 |
| Yun 2021 | 1.30 | 1.09-1.56 | 2.90 | 0.004 | 89 |
| Zhang 2021 | 1.38 | 1.14-1.66 | 3.38 | 0.0007 | 89 |
| Zhang 2020 | 1.30 | 1.09-1.54 | 2.93 | 0.003 | 88 |
| NLR for DCI | | | | | |
| Al-Mufti 2019（1） | 1.72 | 1.22-2.41 | 3.13 | 0.002 | 82 |
| Al-Mufti 2019（2） | 1.74 | 1.24-2.45 | 3.18 | 0.001 | 82 |
| Tao 2017 | 1.63 | 1.20-2.20 | 3.15 | 0.002 | 76 |
| Wu 2019 | 1.91 | 1.60-2.29 | 7.07 | <0.00001 | 0 |
| Yi 2020 | 1.71 | 1.21-2.42 | 3.03 | 0.002 | 79 |
| Zhang 2021 | 1.66 | 1.23-2.24 | 3.29 | 0.001 | 82 |

OR: odds ratio; CI: confidence interval; NLR: neutrophil-to-lymphocyte ratio; DCI: delayed cerebral ischemia.
