# Supplementary material for: The Prognostic Value of Neutrophil-to-Lymphocyte Ratio in Patients With Aneurysmal Subarachnoid Hemorrhage: A Systematic Review and Meta-Analysis of Observational Studies
Source: Front Neurol. 2021 Nov 15;12:745560. doi: 10.3389/fneur.2021.745560 (PMC8636120; doi:10.3389/fneur.2021.745560)
Supplement: Supplementary file 4 [file Data_Sheet_2.DOCX]

Free terms:

(1) neutrophil-to-lymphocyte ratio OR neutrophil to lymphocyte ratio OR neutrophil-lymphocyte ratio OR neutrophil/lymphocyte Ratio OR neutrophil-lymphocyte OR NLR

(2) SAH OR subarachnoid Hemorrhage OR spontaneous Hemorrhage OR ruptured brain aneurysm OR ruptured cerebral aneurysm OR ruptured intracranial aneurysm
